# MHC-linked and un-linked class I genes in the wallaby

**DOI:** 10.1186/1471-2164-10-310

**Published:** 2009-07-14

**Authors:** Hannah V Siddle, Janine E Deakin, Penny Coggill, Elizabeth Hart, Yuanyuan Cheng, Emily SW Wong, Jennifer Harrow, Stephan Beck, Katherine Belov

**Affiliations:** 1 Faculty of Veterinary Science, University of Sydney, NSW 2006, Australia; 2 Wellcome Trust Sanger Institute, Wellcome Trust Genome Campus, Hinxton Hall, Hinxton, Cambridgeshire, CB10 1SA, UK; 3 ARC Centre of Excellence for Kangaroo Genomics, Research School of Biological Sciences, Australian National University, Canberra, ACT 0200, Australia; 4 UCL Cancer Institute, University College London, London WC1E 6BT, UK

## Abstract

**Background:**

MHC class I antigens are encoded by a rapidly evolving gene family comprising classical and non-classical genes that are found in all vertebrates and involved in diverse immune functions. However, there is a fundamental difference between the organization of class I genes in mammals and non-mammals. Non-mammals have a single classical gene responsible for antigen presentation, which is linked to the antigen processing genes, including TAP. This organization allows co-evolution of advantageous class Ia/TAP haplotypes. In contrast, mammals have multiple classical genes within the MHC, which are separated from the antigen processing genes by class III genes. It has been hypothesized that separation of classical class I genes from antigen processing genes in mammals allowed them to duplicate. We investigated this hypothesis by characterizing the class I genes of the tammar wallaby, a model marsupial that has a novel MHC organization, with class I genes located within the MHC and 10 other chromosomal locations.

**Results:**

Sequence analysis of 14 BACs containing 15 class I genes revealed that nine class I genes, including one to three classical class I, are not linked to the MHC but are scattered throughout the genome. Kangaroo Endogenous Retroviruses (KERVs) were identified flanking the MHC un-linked class I. The wallaby MHC contains four non-classical class I, interspersed with antigen processing genes. Clear orthologs of non-classical class I are conserved in distant marsupial lineages.

**Conclusion:**

We demonstrate that classical class I genes are not linked to antigen processing genes in the wallaby and provide evidence that retroviral elements were involved in their movement. The presence of retroviral elements most likely facilitated the formation of recombination hotspots and subsequent diversification of class I genes. The classical class I have moved away from antigen processing genes in eutherian mammals and the wallaby independently, but both lineages appear to have benefited from this loss of linkage by increasing the number of classical genes, perhaps enabling response to a wider range of pathogens. The discovery of non-classical orthologs between distantly related marsupial species is unusual for the rapidly evolving class I genes and may indicate an important marsupial specific function.

## Background

Major Histocompatibility Complex (MHC) class I antigens are responsible for the recognition of pathogenic peptides and form a complex gene family, which can vary in gene number and organization between different species. Although the function of class I genes varies, the structure of the functional molecule nearly always consists of an alpha chain divided into three extracellular domains (α1, α2 and α3), a transmembrane and cytoplasmic region and an associated β2 microglobulin [1].

Class I genes are classified according to function as classical (class Ia) or non-classical (class Ib). Class Ia genes are responsible for pathogen recognition, and perform this function by binding endogenous foreign peptides and presenting them to cytotoxic T-cells on the surface of the cell. Class Ia molecules are ubiquitously expressed and the genes are highly polymorphic within the α1 and α2 domains, where peptide interaction occurs [2], consistent with their role in pathogen recognition. Although the number of class I genes found in different species can vary significantly, the number of expressed class Ia genes in eutherian mammals is reasonably consistent, varying between two to three [3] (the Rhesus macaque is a possible exception [4]). Some non-mammals have only one highly expressed and polymorphic class Ia gene [5,6], while others have multiple class Ia genes [7].

Class Ib genes are related in sequence identity and molecular structure to the class Ia genes, but have lower expression levels, tissue specific expression, low levels of polymorphism and often lack consensus residues important for peptide binding found in class Ia molecules [8]. Human class Ib genes (*HLA-G*, *HLA-E*, and *HLA-F*) do not play a prominent role in presenting antigens to T-cells, but have a variety of roles both related and unrelated to immune function. They interact with natural killer (NK) cells as part of the innate immune response [9,10] and are involved in regulation and suppression of the immune system [11,12]. The number of class Ib genes can vary significantly between species, even between eutherian mammals. Humans have three class Ib genes, while mice have over 30 [13]. Little is known about class Ib genes outside eutherian mammals, however, several class Ib sequences have been described in avians [14], amphibians [15] and one class Ib sequence has been described from a marsupial, the grey short-tailed opossum (*Monodelphis domestica*) (herein referred to as opossum) [16].

Class I genes evolve rapidly through gene duplication and divergence [17]. Due to their rapid evolution, class I genes have undergone species specific expansions and, unless the evolutionary relationship between species is very close (ie human and chimp), orthologous relationships between the class I genes of different species are difficult to detect [18,19]. Human and mouse last shared a common ancestor ~80 million years ago and orthologous relationships between their class I genes are not obvious, even where the function of the genes is homologous.

MHC class Ia genes are found within the MHC region of all species studied to date, however, their organization within the MHC can vary between different species. In non-mammalian species, the MHC class I and class II genes are interspersed with the antigen processing genes (eg TAP) [20-23], which are responsible for transporting peptides from the cytoplasm into the endoplasmic reticulum, where they can be bound by class I molecules. It has been suggested that this ancestral organization of the MHC facilitated the co-evolution of advantageous class Ia and TAP haplotypes that conferred resistance to specific pathogens [20,21]. This organization may have provided a selective advantage to non-mammals with only one class Ia gene as duplication of class Ia would disrupt the co-evolution of TAP and class Ia alleles [21,24]. A single class Ia gene has been identified in the chicken MHC (B locus) [20] and the MHC of *Xenopus *[25]. However, multiple class Ia genes have been described in detail within the quail MHC [7]. It has been suggested that there are multiple class Ia genes in other non-mammalian species, including the axolotl, but number and organization of genes has been estimated without genome sequencing [26].

In the majority of eutherian species the class Ia genes are separated from the TAP genes by the class III region and are interspersed with the framework genes. The rat is an exception and has similarities with non-mammals, as the class Ia genes are linked to antigen processing genes [27]. The framework genes are found within the MHC of all mammals and are highly conserved in gene function and order. It is thought that the repositioning of class Ia genes away from the antigen processing genes and transportation genes prevented the tight co-evolution of class Ia and TAP alleles, but also released constraints on the number of class Ia genes, allowing duplication of class Ia in eutherian mammals [21].

While the class Ia genes are found within the MHC region, class Ib genes have been found outside the MHC in a number of mammalian and non-mammalian species. In *Xenopus *and chicken, class Ib genes have been identified that are not linked to the MHC [15,28]. A more extreme case is that of the class I-like genes *CD1*, which are encoded outside the MHC in mammals and are very divergent in sequence and function to the class Ia and class Ib [29]. The relocation of class Ib genes away from the MHC in these examples is believed to have loosened the constraints on their evolution allowing the genes to evolve independently, without influence of gene conversion events [30] and co-evolution of closely linked genes [24].

Studies have shown that the organization of the marsupial class I genes in the opossum (*Monodelphis domestica*) has similarity to both mammalian and non-mammalian patterns [31]. The class I are interspersed with the antigen processing rather than the framework genes, an organization similar to non-mammals. The opossum has three closely related class I genes that are polymorphic and highly expressed, however, only one class I gene, *Modo-UA*, has been classified as class Ia as the two closely related class I (*Modo-UB *&*Modo-UC*) are located outside the MHC and are not expressed at the same level as *Modo-UA*. The opossum also has six additional putative class Ib genes with unknown functions that are located within the MHC [31]. Class I genes do not localize to a single complex in two marsupials (the opossum and tammar wallaby (*Macropus eugenii*) [31,32]. Two expressed class I genes (*Modo-UB *&*Modo-UC*) are located distal to the MHC at the end of the chromosome in the opossum, while in the tammar wallaby class I genes are MHC un-linked across six different chromosomes.

The tammar wallaby is an Australian marsupial used as a model for studying the unique mode of marsupial reproduction, where young are born after a short gestation period with no immune protection and undergo their development in a pouch. Marsupials are particularly useful for studying the evolution of immune genes in mammals as they last shared a common ancestor with eutherian mammals ~150 million years ago and fill an evolutionary gap in the vertebrate phylogeny between non-mammals and eutherian mammals [33]. A greater understanding of the immune genes of this species will allow comparisons to be made with the opossum, which last shared a common ancestor with the wallaby ~80 million years ago [34] and is the only marsupial for which the MHC has been fully annotated.

Here we report the sequencing of 14 tammar wallaby BACs containing 15 class I genes. Four BACs are located within the MHC proper on chromosome 2q while nine BACs localize to different chromosomes. We show that the class Ib genes remain linked to the MHC on chromosome 2q and are interspersed with the class II and antigen processing genes, while the class Ia genes have MHC un-linked across the genome.

## Results

### Summary of sequenced BACs

**Figure 1:**
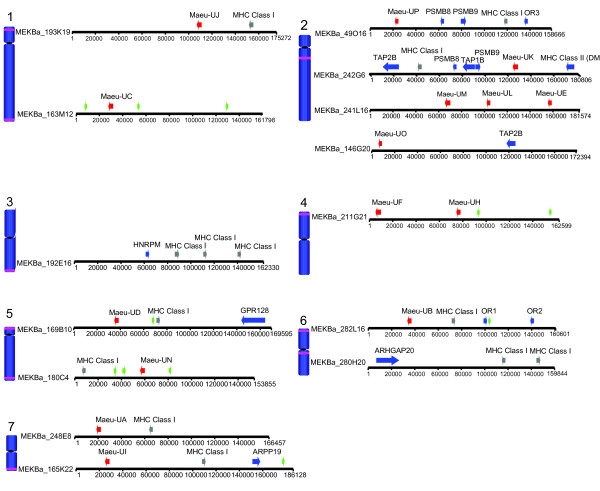
**Schematic diagram of the genomic location and the annotated genes for each sequenced BAC**. The location of each sequenced BAC is indicated by purple stripes on chromosomes. The annotation of the sequenced BAC is shown next to its location. Red arrows indicate class I genes with open reading frames, blue arrows indicate non-class I genes with open reading frames, grey arrows indicate pseudogenes, green arrows indicate KERV sequences. A full list of genes and co-ordinates can be found in Additional file 1.

Fourteen BACs containing class I genes were sequenced and 15 class I genes with open reading frames have been annotated (summarized in Figure 1, Additional file 1 provides a list of genes found on each BAC). The 15 class I genes described in this manuscript are named *Maeu-UA *through to *Maeu-UP *based on the nomenclature for class I genes proposed by Klein and colleagues [35]. The naming scheme does not indicate orthology to other known marsupial class I genes, with the exception of *Maeu-UM *and *Maeu-UK *(see below). The class I genes have 48 – 88.4% amino acid identity to each other across the α1, α2 and α3 domains. The open reading frames of the class I genes vary from 342 to 363 codons.

Previously, we reported the isolation (but not sequencing) of nine tammar wallaby BACs containing class I genes that mapped to every autosome with the exception of chromosome 2, where the MHC class II and class III genes are located [32]. In this study four additional BACs containing class I genes were isolated and localized to the MHC region on chromosome 2. Six class I genes were discovered on the BACs that map to chromosome 2. These genes intersperse with antigen processing genes (*PSMB8*, *PSMB9*, *TAP1B*, *TAP2B*) and the class II gene (*DMB*). Nine class I genes with complete open reading frames were found on the BACs, which map outside the MHC to 9 chromosomal locations.

The BACs which map outside the MHC also contain class I pseudogenes, identified due to frame shift mutations, absence of a start codon, or gene fragmentation, but not other genes that are usually encoded within the MHC. One possible exception is the presence two olfactory receptor genes found on MeKba_282L16 (chromosome 6p) along with *Maeu-UB*. A cluster of olfactory receptor genes is also found adjacent to the framework genes in eutherian mammals and the opossum [31,36], however, further analyses of these genes is required to determine the relationship of these olfactory receptors to the MHC-linked olfactory receptor genes in other species.

### There are two distinct groups of class I genes in the tammar wallaby

The wallaby class I genes form two distinct groups, MHC-linked class I genes (*Maeu-UE*, *UL, UK, UM, UO *and *UP*) and MHC un-linked class I genes (*Maeu-UA, UB, UC, UD, UF, UH, UI, UJ *and *UN*). The MHC un-linked class I genes have greater amino acid identity to each other than to the six class I genes linked to the MHC, sharing on average 85% nucleotide identity with each other and 63% identity to the MHC-linked class I genes. There is one exception: *Maeu-UP*, located on chromosome 2, shares 72–75% nucleotide identity with the MHC un-linked class I genes and 61–67% identity to the MHC-linked class I genes. The MHC-linked class I genes share features of their amino acid sequence, including a deletion of varying size in the α1 domain between residues 59 and 66 (Additional file 2: Amino acid alignment of tammar wallaby class I genes, opossum class I genes and *HLA-A*) and a short intron between the α1 and α2 domains (data not shown), compared to the MHC un-linked class I genes.

**Figure 2:**
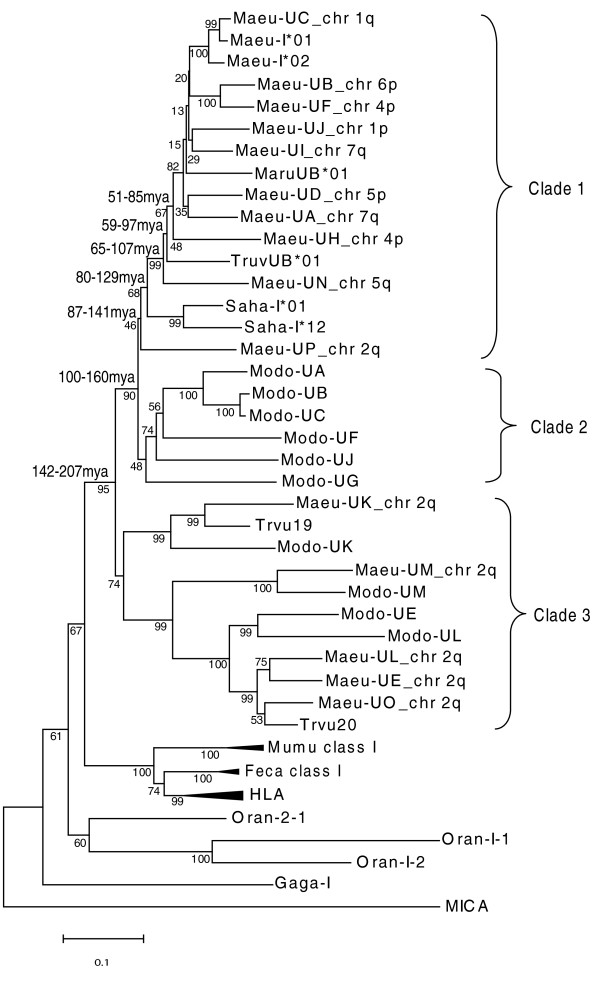
**Neighbour-joining phylogenetic analysis of the relationship between marsupial class I genes**. Bootstrap support for branches is provided. Estimated divergence times are indicated adjacent to the relevant node. The tammar wallaby class I sequences Maeu-I*01 and Maeu-I*02 are cDNA transcripts from Genbank. The phylogenetic tree is divided into the following clades; clade 1 includes MHC un-linked wallaby class I genes and expressed class I transcripts from Australian marsupials, clade 2 includes opossum class I genes only, including opossum class Ia (*Modo-UA, UB *and *UC*), clade 3 includes marsupial specific class I genes from both the American and Australian lineages.

The MHC un-linked class I genes group together (clade 1), in a phylogenetic analysis, while MHC-linked class I genes on chromosome 2q form a separate and distinct clade (clade 3) (Figure 2). Again, *Maeu-UP *is an exception, occupying the basal position in clade 1. Although the position of *Maeu-UP *is not well supported using neighbour joining (Figure 2) or Bayesian analysis (Additional file 3: Phylogenetic tree showing divergence times for tammar wallaby class I genes and Additional file 4: Phylogenetic tree produced for BEAST analysis) both methods produced trees with the same topology. Clade 1 also contains previously identified expressed class I transcripts from the Australian marsupials including the red-necked wallaby (*Macropus rufogriseus*), brushtail possum (*Trichosurus vulpecula*), Tasmanian devil (*Sarcophilus harrisii*) and tammar wallaby. Opossum class I sequences, including the *Modo-UA *(class Ia) and *Modo-UG *(class Ib) form a sister clade (clade 2) to the MHC un-linked tammar wallaby class I genes. Interestingly, clade 3 contains the tammar wallaby MHC-linked class I genes (*Maeu-UE, Maeu-UO, Maeu-UK, Maeu-UM *and *Maeu-UL*), two class I sequences isolated from the brushtail possum(*Trichosurus vulpecula*) and the majority of the opossum class Ib sequences. This clade is basal to the opossum class Ia sequences and MHC un-linked tammar wallaby class I sequences.

### The MHC-linked class I genes have characteristics of non-classical class I genes

**Figure 3:**
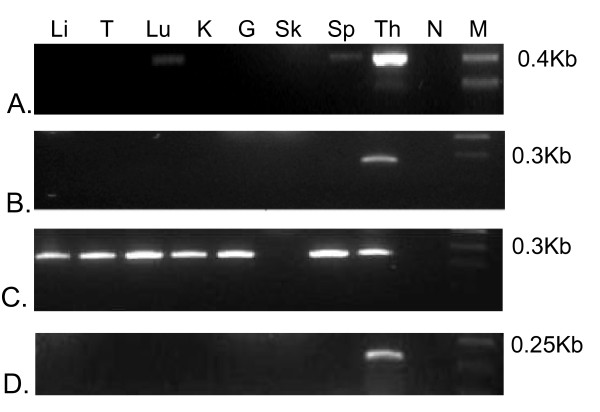
**Expression profiles of MHC-linked class Ib genes**. A. *Maeu-UM*, B. *Maeu-UE*, C. *Maeu-UK*, D. *Maeu-UO*. The tissues tested are given as; Li – liver, T – testis, Lu – lung, K – kidney, G – gut, Sk – skin, Sp – spleen, Th – Thymus, N – negative, M – marker. The length of the amplified cDNA fragment is given next to the marker.

Six MHC-linked class I genes were identified, *Maeu-UK, Maeu-UM, Maeu-UE*, *Maeu-UL, Maeu-UP *and *Maeu-UO*. Four of these genes are expressed and have characteristics of class Ib genes, *Maeu-UK, Maeu-UM, Maeu-UE *and *Maeu-UO*. Gene specific primers were used to amplify each of these genes from the wallaby thymus and no more than two alleles was amplified for each primer set, suggesting the primers are gene specific. No expression was detected for *Maeu-UP *or *Maeu-UL*. Three of the MHC-linked class I genes were found to have tissue specific expression, *Maeu-UM, Maeu-UE *and *Maeu-UL*. Expression of *Maeu-UM *was found in wallaby lung, spleen and thymus, and transcripts of *Maeu-UE *and *Maeu-UO *were only detected in the thymus (Figure 3). *Maeu-UK *was widely expressed, found in all tissues examined, with the exception of skin (liver, testes, lung, kidney, gut, skin spleen and thymus tissue (Figure 3).

*Maeu-UK, Maeu-UM, Maeu-UE *and *Maeu-UO *sequences were amplified from four wallabies of Kangaroo Island origin (I1, I2, I3 and I4. Again, no more than two alleles per gene were amplified from each individual (Table 1). The amplified alleles of *Maeu-UK, Maeu-UM, Maeu-UE *and *Maeu-UO *lacked variation at the peptide binding region with only a maximum of one non-synonymous substitution between the identified alleles of each gene. Comparison of the level of polymorphism in these genes with the only other characterized marsupial class Ib, *Modo-UG*, revealed a similar level of polymorphism (Table 1).

**Table 1:** Number of alleles for each Class Ib locus and number of polymorphic sites within the peptide binding region (PBR)

**Locus**	**Number of alleles/** **No. of individuals**	**No. polymorphic sites within PBR**
*Maeu-UM*	3/4	1

*Maeu-UE*	2/4	0

*Maeu-UK*	3/4	1

*Maeu-UO*	2/4	0

*Modo-UG*	12/16	2

### MHC-linked class I genes in the tammar wallaby appear marsupial specific

The topology of the phylogenetic tree clearly separates the opossum and Australian marsupial class I sequences of clades 1 and 2 from the class I sequences in clade 3. Rather than forming species specific clades, as is the norm of class I genes, the class I genes in clade 3 include class Ib genes of the tammar wallaby, brushtail possum and the opossum. Within this clade, two wallaby class I genes *Maeu-UM *and *Maeu-UK *are clearly orthologous to the opossum class Ib genes, *Modo-UM *(bootstrap of 100) and *Modo-UK *(bootstrap of 100). No orthologs for *Modo-UE *and *Modo-UL *were discovered in the wallaby, however, three closely related class Ib genes, *Maeu-UE*,*UL *and *UO *were identified and cluster with *Modo-UE *and *Modo-UL *in clade 3. One of these genes, *Maeu-UO*, does have an orthologue in the brushtail possum.

### The MHC un-linked class I genes have characteristics of classical class I

Nine MHC un-linked class I genes were found throughout the genome, three of these genes have characteristics of class Ia genes. Evidence of expression was found for seven of these genes, while two are either pseudogenes or have such low levels of expression that transcripts were not detected. A number of lines of evidence indicate that seven of these genes are expressed and that three are highly expressed and have a classical function. First, class I transcripts sequenced from blood and thymus samples identified from a number of individuals are most similar to MHC un-linked class I genes *Maeu-UA *(chr 7)*, Maeu-UB *(chr 6)*, Maeu-UC *(chr 1)*, Maeu-UD *(chr 5)*, Maeu-UF *(chr 4), *Maeu-UH *(chr 4) and *Maeu-UN *(chr 5) in a phylogenetic tree (Additional file 5: Phylogenetic relationship between expressed class I transcripts and genomic class I genes). Between 7 and 13 sequences were isolated from every individual, indicating that at least seven class I genes are amplified, although assignment of sequences to loci was difficult due to high levels of sequence similarity and a level of polymorphism observed in the peptide binding region. The majority of transcripts sequenced from the blood and thymus samples were most similar to *Maeu-UA, Maeu-UB *and *Maeu-UC*. Class Ia genes have high levels of expression compared to class Ib genes and are more easily isolated from the transcriptome, thus the class I transcripts most readily isolated are most likely to be the class Ia genes. No transcripts showing high similarity to *Maeu-UI *and *Maeu-UJ *were identified suggesting that these loci may not be expressed. However, it cannot be ruled out that these genes are expressed at very low levels and not detected here. Transcripts similar to the class Ib genes on chromosome 2 are not amplified with these primers. Second, previously identified expressed class I genes isolated from Australian marsupials, including the red-necked wallaby, possum and Tasmanian devil, have a close relationship to the MHC un-linked wallaby class I genes. The majority of these class I genes are thought to be class Ia genes, in particular Saha-I*01 [37,38]. Finally, the promoter elements of the MHC un-linked class I genes are identifiable and highly similar in sequence identity to the promoter regions of class Ia genes in the opossum (*Modo-UA*) and eutherian mammals, while the promoters of the putative class Ib genes are more divergent (see detailed analysis below).

### Promoter elements of class I genes

**Figure 4:**
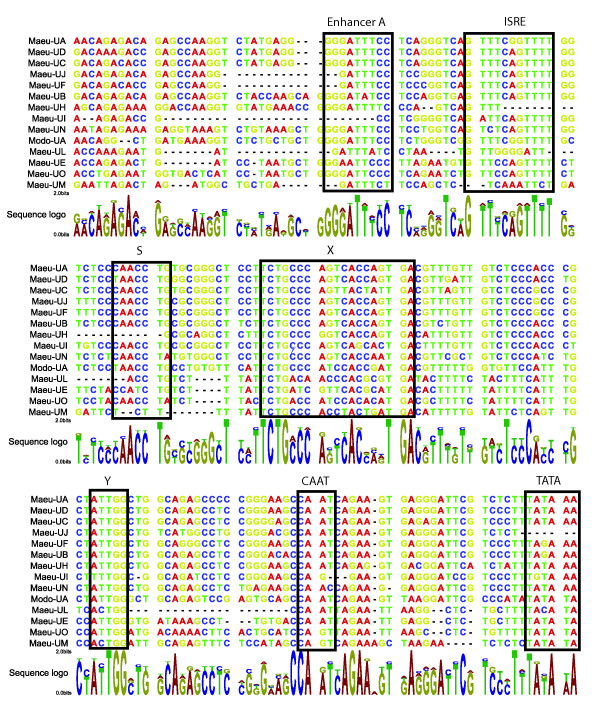
**Promoter elements of MHC-linked and MHC-unlinked class I genes**. 180 base pairs of sequence upstream from the TATA box is shown for all wallaby class I genes, with the exception of *Maeu-UK *and *Maeu-UP*, for which promoter elements could not be identified. The promoter elements for *Modo-UA *are also included. The boxed elements are, Enhancer A, an IFN stimulated response element (ISRE), S, X and Y motifs, a CAAT element and TATA box within 50 base pairs of the start site for each gene.

Putative class I promoter elements were identified within 200 base pairs of the start codon for the class I genes and included TATA and CAAT sites, the S, X and Y regulatory motif, an interferon stimulated response element (ISRE) and an enhancer A site (Figure 4). The promoter elements of the MHC un-linked class I genes are highly similar to each other, sharing between 66–95% nucleotide identity in the 290 base pairs upstream of the gene start site. The promoter elements of the MHC un-linked class I are also similar to the regulatory elements of *Modo-UA*, sharing 57–70% nucleotide identity. However, *Maeu-UJ *is missing a TATA site and appears to have a truncated enhancer A element and *Maeu-UH *and *Maeu-UI *have no enhancer A element. These variations may account for the lack of evidence of transcription of *Maeu-UJ *and *Maeu-UI*. The upstream sequence of the class Ib, MHC-linked class I genes shared between 32–50% nucleotide identity with the MHC un-linked class I, 35–79% with one another and only 39–51% with the promoter sequence of *Modo-UA*, consistent with the more divergent nature of the coding sequence of these genes. However, it was still possible to identify the TATA, CAAT and SXY motifs in these genes, with the exception of *Maeu-UK *and *Maeu-UP*. Putative enhancer A and ISRE elements of the class Ib genes were identified with less confidence due to the lower sequence identity in the upstream sequence observed for these genes.

### Identification of Kangaroo Endogenous RetroViral (KERV) elements

Sequences with high similarity to Kangaroo Endogenous RetroViral (KERV) elements were identified on six of the BACs. One full length KERV sequence was identified on chromosome 5p, adjacent to *Maeu-UN*. This KERV sequence had 90.6% nucleotide similarity to a previously described full length KERV (KERV-1) [39]. Numerous partial KERV sequences were identified at 1p, 3q, 4p, 5q and 6p and 7p (shown in Figure 1). The partial KERV sequences varied in length from 290 base pairs to 514 base pairs and shared between 67% and 99% nucleotide identity to the full length KERV on chromosome 5p. No significant matches to KERV were found within the BACs on chromosome 2.

### Molecular dating of class I gene relocation

Molecular dating was used to determine approximate timing of the repositioning of class I genes away from the MHC in the tammar wallaby. The molecular dating performed in this study is treated as an estimate only as it does not take into account that MHC class I and class II genes evolve at a faster rate than neutral evolution would predict, due to balancing selection [40]. *Maeu-UP *is located within the MHC on chromosome 2 and is the most similar MHC-linked class I in sequence identity to the MHC un-linked class I genes. It is found at the base of clade 1, which contains putative classical class I sequences. Using a Bayesian approach for molecular dating (see Methods), the MHC un-linked class I genes and *Maeu-UP *last shared a common ancestor ~86–140 mya. *Maeu-UN *on chromosome 5q3, the most basal MHC un-linked class I gene was the first class I gene to move away from chromosome 2, ~65–107 mya. The subsequent duplication of class I genes and their repositioning to different chromosomal positions most likely occurred from this location with the most recent duplications and relocation occurring 15–38 mya.

## Discussion

The organization of the Major Histocompatibility Complex of the wallaby is unique among mammals and provides a model for understanding how genomic location affects gene function, co-expression and co-evolution. The wallaby has 15 class I genes, 11 of which are transcribed. This figure is similar to the opossum, which has 11 class I genes, 9 of which are transcribed [31]. Of the 15 class I genes with open reading frames, three are putative class Ia genes *(Maeu-UA *(chr7)*, Maeu-UB *(chr6) and *Maeu-UC *(chr1)), four are putative class Ib genes (*Maeu-UM, Maeu-UK, Maeu-UE *and *Maeu-UO*), four show no evidence of expression and the remaining genes appear to be expressed only at low levels and are not classified as class Ia or class Ib (*Maeu-UD, Maeu-UF*, *Maeu-UH *and *Maeu-UN*). The number of class Ia genes in the tammar wallaby is similar to eutherian species (usually 2 or 3).

It is likely that the tammar wallaby class Ia genes were originally located within the MHC on chromosome 2, interspersed with antigen processing genes. The first repositioning of wallaby class I genes away from chromosome 2 led to the class I gene, *Maeu-UN*, at the telomeric end of chromosome 5q, given its basal position on the phylogenetic tree (Figure 2). Molecular dating indicates that this repositioning occurred over 65 mya and that all the class Ia and class Ia-like genes trace back to this common ancestor. *Maeu-UP*, located on chromosome 2, most likely gave rise to *Maeu-UN *as it is the class I gene most closely related to the MHC un-linked class I genes. We previously proposed that the Kangaroo Endogenous RetroViral elements (KERV), found at the centromeres of all tammar wallaby chromosomes and near the telomere of chromosome 5, may have played a role in the repositioning of class I genes by creating instability in the MHC region (which is located near a KERV-rich area at the centromere of chromosome 2) [32,41]. The results of this study confirm the presence of partial KERV sequences located adjacent to a number of the MHC un-linked class I genes (Figure 1) and provides support for the hypothesis that KERVs may have contributed to the duplication and movement of class I genes in the wallaby. All MHC un-linked class I genes described here are located at subtelomeric or pericentric regions, which are known hotspots not only for segmental duplication [42], but also the insertion and retention of repeat sequences, illegitimate recombination and frequent rearrangements [43]. In the wallaby there is evidence of subtelomeric and pericentric rearrangement of genes. Three genes, *ARHGAP20, GPR128 *and *HNRPM*, located adjacent to MHC un-linked class I genes in the wallaby are in unexpected positions when compared to their homologues in the opossum genome and based on karyotype evolution of marsupials or gene mapping data (Table 2) [44,45]. These movements suggest these regions have been subject to rearrangement in the wallaby.

**Table 2:** Non-MHC genes on MHC un-linked Class I BACs and their predicted location in the wallaby

**Gene**	**BAC location in wallaby**	**Location in opossum**	**Predicted location in wallaby**
*HNRPM*	3	3	4 [45]

*GPR128*	5q (distal)	4	5q (proximal) [44]

*ARPP19*	7	1	7 [45]

*ARHGAP20*	6	4	5 [45]

Retrotransposons can act as recombination hotspots in the genome via homologous recombination between retrotransposon sequences MHC un-linked on different chromosomes [46]. Phylogenetic analysis shows that wallaby class I genes on different chromosomes may be more closely related to one another than to an adjacent class I (eg *Maeu-UB*-6q and *Maeu-UF*-4p). A similar pattern emerges when the KERV fragments on different chromosomes are compared and may indicate that the class I genes and KERVs are causing recombination hotspots within the genome, fueling class I gene diversification and polymorphism, and providing an advantage for the wallaby MHC. The presence of numerous partial KERVs suggests these elements have not been recently active and that the original movement of a class I gene to chromosome 5 (perhaps by amplification of KERV and transposition) occurred early in the evolution of the Australian marsupial lineage, which is supported by the predicted date of movement, approximately 65 mya.

The repositioning of class Ia genes out of the MHC in wallaby ancestors over 65 million years ago indicates that class I genes are located outside the MHC in many other Australian species, including other macropods and the brushtail possum, as these species diverged less than 30 million years ago [34]. Previously we suggested that *Modo-UB *and *Modo-UC *were evolving towards class Ib genes due to their location outside the MHC [31]. Given that wallabies have non MHC-linked class Ia genes, it is feasible that *Modo-UB *and *UC *are also classical given their close relationship to *Modo-UA*. The presence of class Ia genes, responsible for antigen presentation, outside the MHC of marsupials for an extended period of evolutionary time suggests an advantage is gained in having class Ia genes organized in this way.

It has been suggested that the repositioning of class Ia genes away from the antigen processing genes in mammals allowed the class Ia genes to duplicate [21]. We propose that marsupials gained a similar advantage from moving class Ia genes outside the MHC. Some non-mammals have only one class Ia gene, which in species such as *Xenopus *[21], chicken [20] and nurse shark [22] is linked to the antigen processing genes within the MHC. This ancestral MHC organization has the advantage that TAP and class Ia alleles co-evolve [5]. For instance, the chicken TAP genes are polymorphic, with specific TAP alleles transporting peptides for specific class Ia alleles, providing an efficient response to certain pathogens [20]. Duplication and subsequent diversification of such class Ia genes would therefore disrupt the advantageous linkage and resulting co-evolution between class Ia and TAP alleles [21,24]. Co-evolution of class I and TAP is not confined to non-mammals, but has been observed within the rat MHC as well [47]. However, in the majority of eutherian mammals the class Ia genes have moved away from the TAP genes to become interspersed with the framework genes of the MHC. The TAP genes are no longer polymorphic, but have become promiscuous and can present a broader array of pathogenic peptides [24]. Marsupials and eutherian species, independently, found it advantageous to separate class Ia genes from antigen processing genes, resulting in differing class I organization between these lineages of mammals, but effectively the same outcome, multiple class Ia genes. However, it should be noted that some non-mammalian species have multiple class Ia genes adjacent to TAP genes and may have diversified class I genes, while maintaining linkage with antigen processing genes [7].

The extensive duplication and dispersal of class I genes in the wallaby may have been assisted by their complete removal from the MHC and the constraints this region imposes. In eutherian mammals the class I genes are interspersed with the framework genes, which are highly conserved. The duplication and repositioning of class I genes in this region is believed to be restricted by these framework genes, as class I movement that disrupts these genes would be deleterious [48]. In the wallaby the class I genes have moved away from the MHC altogether and their duplication and repositioning is not restricted. Of the nine MHC un-linked class I genes, not all are expressed at the same level and three appear highly expressed and have evidence of polymorphism. Thus, there is still a restraint on the maximum number of class Ia genes due to the need to maintain a wide spectrum of T cells after thymic selection.

The MHC-linked class I genes in the tammar wallaby are non-classical class I genes, based on promoter elements, expression patterns and levels of polymorphism. The changes in promoter sequence of the class Ib genes in the tammar wallaby perhaps explains the variation in expression patterns for these genes. *Maeu-UM, UE*, and *UO *have tissue-specific expression and are not polymorphic, with few substitutions in the PBR of different alleles. *Maeu-UK *has low levels of polymorphism and is expressed in all tissues. *Maeu-UM *and *Maeu-UK *are clear orthologs of the opossum class Ib genes, *Modo-UM *and *Modo-UK*, despite these species being separated by 80 million years of evolution and belonging to different Orders. An expressed transcript has been isolated from the brushtail possum (Trvu19) that is closely related to *Maeu-UK *and *Modo-UK *[49]. This transcript may represent a processed pseudogene as it has a frameshift mutation at the 5' end of the α1 domain [49]. However, expressed transcripts from the tammar wallaby show no evidence of this mutation and also include the leader peptide. Further, the frameshift mutation described in the possum sequence is very close to the forward primer site used to amplify this sequence. It is possible that the described mutation is a sequencing artifact, as conservation of a processed pseudogene in the possum is unlikely. As there is evidence that an ortholog of *UK *exists in the brushtail possum we predict all Australian marsupials would share the *UK *class I gene. Orthology between class I genes of distantly related species is highly unusual for class I genes, which typically form species specific clades due to their rapid evolution [50]. It has been proposed that MHC polymorphism can be passed to species as they evolve from a common ancestor [51], however, no examples of class I orthology have been demonstrated between species belonging to different phylogenetic orders [18,50]. The class I genes of human and mouse, separated by a similar evolutionary distance as the tammar wallaby and opossum, do not show clear orthology, despite some genes, such as *HLA-E *and *H2-Qa1*, sharing similar functions. It is believed their orthologous relationship is obscured by gene conversion [30]. The conservation of orthologs, *Modo-UM/Maeu-UM *and *Modo-UK/Maeu-UK*, over such an extended period of time leads us to speculate that these genes may have a critical, marsupial-specific function. The marsupial mode of reproduction and development poses immunological challenges to the pouch young, which are born after a short gestation and are immuno-naïve at birth. We propose that conserved marsupial class Ib molecules may play a role in the immunological protection of marsupial young. *Maeu-UM *is expressed in the lung as well as the spleen and thymus, and it is possible this molecule plays a role in the protection of exposed surfaces. Ongoing studies in our laboratories will look into the function of these molecules in a range of marsupials.

## Conclusion

The tammar wallaby has a novel MHC class I organization, which has successfully maintained functional, diverse class I genes. The class I genes do not follow conventional models of class I evolution as class Ib genes have remained linked to the MHC whereas the class Ia genes are not linked to the MHC but have maintained polymorphism and high expression levels. This unique organization provides a model for studying the importance of MHC gene clustering on gene function. Furthermore, our analysis has shown that some marsupial class Ib genes are remarkably conserved within this highly adapted mammalian lineage. The important biological differences between marsupial and eutherian mammals in their mode of reproduction and the pathogenic pressures on their immune system can be used to provide insights into how class Ib function is adapted to the biology of a species.

## Methods

### BAC selection and sequencing

We have previously described the isolation and physical mapping of nine of the class I BACs described here from a 10× tammar wallaby BAC library (Arizona Genomics Institute) to chromosomes 1, 3, 4, 5, 6, and 7 [32]. An additional four class I BACs have now been isolated and physically mapped to chromosome 2q according to Deakin et al. (2007) [32]. All BACs were confirmed to contain class I genes by southern blot analysis using a previously described conserved class I exon 4 fragment as a probe [52]. This analysis was also used to determine which BACs were overlapping based on shared bands and redundant BACs were excluded from the sequencing process. In total, 14 BACs were selected, sequenced and annotated as previously described [36] and submitted under the following accession numbers to the Genbank database: MEKBa_248E8 [CU457750]; MEKBa_211G21 [CU302408]; MEKBa_242G6 [CU463018] MEKBa_146G20 [CU466525] MEKBa_49O16 [CU463996]; MEKBa_241L16 [CU463962]; MEKBa_282L16 [CU302415]; MEKBa_169B10 [CU302406]; MEKBa_180C4 [CU302373]; MEKBa_192K19 [CU302374]; MEKBa_165K22 [CU302372]; MEKBa_280H20 [CU302409]; MEKBa_192E16 [CU302407]; MEKBa_163M12 [CU302420].

### Phylogenetic and sequence analysis of class I genes

The predicted, full length coding sequences of 15 class I genes were aligned with the following class I sequences from the NCBI database using ClustalW [53] with some manual adjustment: Tammar wallaby: Maeu-1*01, DQ304109; Maeu-1*02, DQ304110; Red-necked wallaby: Maru UB*01, L04952; Brushtail possum: Trvu UB*01, AF359509; Trvu19, EU570828; Trvu20, EU570829; Tasmanian devil: Saha-I*01, EF591089, Saha-I*12, EF591100 & Saha-I*13, EF591101; Opossum: Modo UA*01, AF1255540, Modo UB*01, AF522352, Modo UC*01, AF522352, Modo-UF, Modo-UG, Modo-UJ, Modo-UE, Modo-UL, Modo-UM & Modo-UK can be found at ; Human: HLA-Cw*1203, U06487, HLA-B*0701, U21052 &; Mouse: Mumu H-2D^b^, U47325, Mumu H-2D^d ^U47326 & Mumu H-2K^b ^U47328; Platypus: Oran ClI, AY112715 (Oran-2-1), Oran-1-1, EU030443 (ABU86900.1), Oran-1-2,, EU030443 (ABU86901.1); Chicken: Gaga B-FIV, AF013491; Human: Hosa MICA, L14848. The nucleotide and amino acid identity between the sequences was calculated using Bioedit [54]. A neighbour joining tree was constructed with the nucleotide sequence of the α1, α2 and α3 domains (across 654 nucleotides) using Jukes-Cantor distance [40,55] with 1000 bootstraps in the Mega 4.0 software [56]. The phylogenetic analysis was repeated with the α3 domain only and the α1 and α2 domains only to test for differences in relationships between different regions of the genes, but the topology of the tree was not altered.

Molecular dating of genes was accomplished using BEAST [57]. Calibration nodes were defined using divergence dates for tammar wallaby and opossum; brushtail possum and tammar wallaby; and block duplication dates of human HLA-C and HLA-B. The Yule process was used as tree prior. Three identical analyses were run to check for consistency between runs. Chain length was set to 10,000,000 and logged every 1,000 steps. Tracer [57] was used to analysis BEAST output. Trees were summarized using a burning value of 1,000. A maximum clade credibility summary tree was produced where node heights equaled mean heights. FigTree was used to produce the final tree.

Promoter sequences were identified by performing a dot plot analysis [58] on the upstream nucleotide sequence (2000 bp) of *Modo-UA *and the wallaby class I genes to identify conserved regions of the sequence. Specific elements were then identified manually by aligning the wallaby class I genes to *Modo-UA*, mammalian (pig) and non-mammalian (duck) class I promoters using ClustalW. Nucleotide identity between promoter sequences were calculated as described above.

KERV sequences were identified using BLAST (Basic Local Alignment Search Tool) with a previously identified full length KERV (KERV-1, Genbank-AF044909) sequence as the query. KERV sequences were extracted and aligned with KERV-1 using ClustalW.

### Primer design

Gene specific forward and reverse primers were designed to amplify the α1 and α2 domains of the MHC-linked class I genes, *Maeu-UE *(Primer set 1), *Maeu- UK *(Primer set 2) *Maeu-UO *(Primer set 3) and *Maeu-UM *(Primer set 4) (Table 3). The forward primers were designed as close to the beginning of the α1 domain as possible and the reverse primers were designed as close to the end of the α2 domain as possible. Variation in the primer sites was necessary to maintain specificity for each gene. The nine MHC un-linked class I genes show a high level of nucleotide identity, thus the design of locus specific primers for these genes was impossible. Instead, previously described tammar wallaby class I cDNA sequences isolated from the spleen [52] were used to design multi-locus primers that amplify the α1, α2 and α3 domains of the MHC un-linked class I genes (primer set 5).

**Table 3:** Primers used for class I gene amplification. Class I loci for which no expression was detected *(Maeu-UP *and *Maeu-UL*) were excluded.

**Class I locus**	**Primers**
*Maeu-UE*	α1F – ATG TGC CTG CAG AAA GTG TCT GCG
	
Primer set 1	α2R – AT GGT TCA GGG CTC CTG AGT TCC

*Maeu-UK*	α1F – AGT AGT TAG AGA GAC GGA GCA CAC
	
Primer set 2	α2R – GTA CTT CTG CAG CCA TTC AGT

*Maeu-UO*	α1F – CAA GAG ATA CCA GAT TAC TGG GA
	
Primer set 4	α2R – CGT TCC CAG CGA TCC AAC TTA GA

*Maeu-UM*	α1F – GCG GGC CCA GAC TGG GGT TAG AG
	
Primer set 4	α2R – ACG TTT AGG GCC ACG TTG TCC AAT

*MHC un-linked class I*	α1F – CACTCCATGAGGTATTTCGACA
	
Primer set 5	α3R – GGCTCAGGCAGCCCCTCGTGC

### Expression studies on MHC un-linked class I genes

RNA was extracted from the blood of four individuals (I–IV) (Tri-reagent BD, Sigma-Aldrich) and reverse transcribed (Superscript III, Invitrogen). RNA was extracted from the thoracic thymus of a fifth individual (V) and 5' RACE cDNA was made (GeneRacer, Invitrogen). To determine which of the nine MHC un-linked class I genes are expressed two approaches were used. First, primer set 5 was used to amplify the α1, α2 and α3 domains of expressed class I genes from the blood of individuals I–IV using high fidelity taq polymerase (Platinum DNA taq polymerase high fidelity, Invitrogen) and the following PCR conditions: Initial denaturation at 94.0°C for 3 min, followed by 29 cycles of 94.0°C for 30 s, 60°C for 30 s, and 72°C for 2 min, and a final extension at 72°C for 10 min. Twenty-five clones were sequenced from individuals I–IV, a total of 125 clones.

Second, to ensure all potential transcripts were isolated, the α3 reverse primer from primer set 6 and 5' GeneRacer primer (Invitrogen) were used to amplify expressed class I from the thymus sample of individual V with the following PCR conditions: Initial denaturation at 94.0°C for 3 min, followed by 29 cycles of 94.0°C for 30 s, 68°C for 30 s, and 72°C for 2 min, and a final extension at 72°C for 10 min. The resulting PCR products were cloned into the pGEM-T easy vector system (Promega) and 25 clones were sequenced in both directions. All sequences were quality checked in Sequencher 4.1.4 (GeneCodes) and all unique class I transcripts were compared to the genomic class I genes by constructing a neighbor joining tree with 1000 bootstraps in Mega 4.0. The resulting phylogenetic tree was used to determine class I transcripts highly similar to genomic class I genes and these class I transcripts have been submitted to Genbank with the accession numbers FJ238079 to FJ238088.

### Expression studies on MHC-linked class I genes

Gene specific primer sets (1–4), described above, were used to amplify *Maeu-UE, Maeu-UO, Maeu-UP, Maeu-UM*, and *Maeu-UK *from the thymus RACE cDNA of individual V, to determine if these genes are expressed. The PCR conditions for each gene showing evidence of expression were as follows: *Maeu-UM *– Initial denaturation at 94.0°C for 3 min, followed by 29 cycles of 94.0°C for 30 s, 63°C for 30 s, and 72°C for 1 min, and a final extension at 72°C for 10 min; *Maeu-UK*, *Maeu-UE*, *Maeu-UO *– Initial denaturation at 94.0°C for 3 min, followed by 29 cycles of 94.0°C for 30 s, 60°C for 30 s, and 72°C for 1 min, and a final extension at 72°C for 10 min. The amplified products varied between 0.25 kb and 0.4 kb, depending on the primer set. The amplified products were cloned into the pGEM-T easy vector system (Promega) and six clones were sequenced in both the forward and reverse directions for each gene. No more than two alleles were amplified for each primer set. The sequences were quality checked in Sequencher 4.1.4 (GeneCodes) and aligned with the class I genes annotated on the BACs. All transcripts have been submitted to Genbank with the accession numbers FJ238064, FJ238067, FJ238071 and FJ238075.

Expression of *Maeu-UE, Maeu-UO, Maeu-UM*, and *Maeu-UK *was detected in the thymus. No evidence of expression was detected for *Maeu-UL *or *Maeu-UP *despite a range of PCR conditions trialed, so PCR conditions for these primer sets are not described here and further expression studies were not performed. The expression pattern of *Maeu-UE, Maeu-UO, Maeu-UM*, and *Maeu-UK *was then assessed in the spleen, testis, liver, lung, kidney and gut. RNA was extracted from the tissues of a single individual (Tri-reagent, Sigma-Aldrich) and reverse transcribed (Superscript III, Invitrogen). The RNA was not treated with DNase to enable the intron between exon 2 and exon 3 to be amplified and compared between different genes. Primer sets 1, 3, 4 and 5 were used to test for expression of each gene.

### Polymorphism studies on MHC linked class I genes

Primer sets 1–4 were used to amplify *Maeu-UE, Maeu-UO, Maeu-UM *and *Maeu-UK *from DNA of individuals I–V using the PCR conditions described above. *Maeu-UL *and *Maeu-UP *were excluded as expression was not detected for these genes (see below). The resulting fragment included exon 2, exon 3 and the intervening intron. The amplified fragments were cloned into a pGEM-T easy vector (Promega) and six clones from each individual were sequenced in both directions. The sequences were quality checked in Sequencher 4.1.4 (GeneCodes) before being imported into bioedit and compared for polymorphism. No more than two alleles were amplified for each gene. The unique alleles for each gene have been submitted to Genbank with the following accession numbers FJ238065, FJ238066, FJ238068, FJ238069, FJ238070, FJ23807, FJ238073, FJ238074, FJ238076 and FJ238077.

## Authors' contributions

KB and SB designed the project. HVS isolated BACs, carried out phylogenetic and sequence analysis of class I genes and promoters, carried out polymorphism and expression studies and drafted the manuscript. JED isolated BACs, carried out FISH and isolated KERV sequences. PC fingerprinted BACs. EH and JH annotated BACs. YC assisted with polymorphism studies. ESWW carried out molecular dating. All authors edited and approved final manuscript, with particular help from KB and JED.

## Supplementary Material

Additional file 1 **Table of all annotated genes**. The co-ordinates of all genes and pseudogenes annotated on each BAC. Click here for file

Additional file 2 **Amino acid alignment of tammar wallaby class I genes, opossum class I genes and HLA-A**. Amino acid alignment, the following features are marked, P – residue involved in peptide binding, * – glycosylation site, c- cysteine residues forming disulphide bridges, a dash indicates a deletion in some tammar wallaby and opossum class Ib genes. Click here for file

Additional file 3 **Phylogenetic tree showing divergence times for tammar wallaby class I genes**. Phylogenetic tree produced for the BEAST analysis with all divergence estimates and confidence belts in blue. Click here for file

Additional file 4 **Phylogenetic tree produced for BEAST analysis**. Phylogenetic tree produced for the BEAST analysis with posterior distribution values, 1 = 100%. Click here for file

Additional file 5 **Phylogenetic relationship between expressed class I transcripts and genomic class I genes**. Neighbour joining phylogenetic tree comparing class I transcripts isolated from the tammar wallaby thymus and blood to genomic class I genes from BACs. Click here for file
