# Supplementary figures and images for: MHC-linked and un-linked class I genes in the wallaby
Source: BMC Genomics. 2009 Jul 14;10:310. doi: 10.1186/1471-2164-10-310 (PMC2719672; doi:10.1186/1471-2164-10-310)

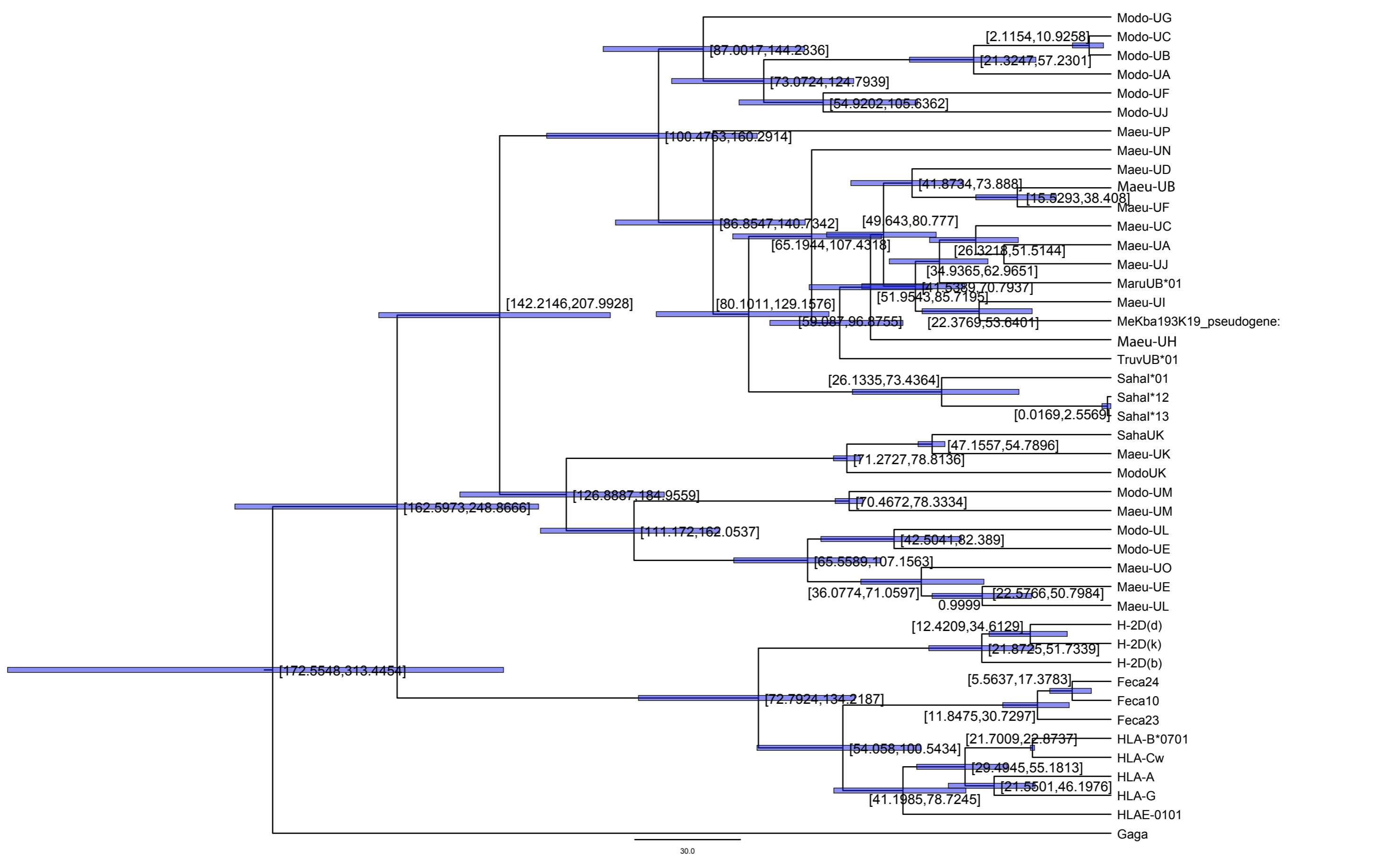

Supplement: Additional file 3 — Phylogenetic tree showing divergence times for tammar wallaby class I genes. Phylogenetic tree produced for the BEAST analysis with all divergence estimates and confidence belts in blue. [file 1471-2164-10-310-S3.pdf]

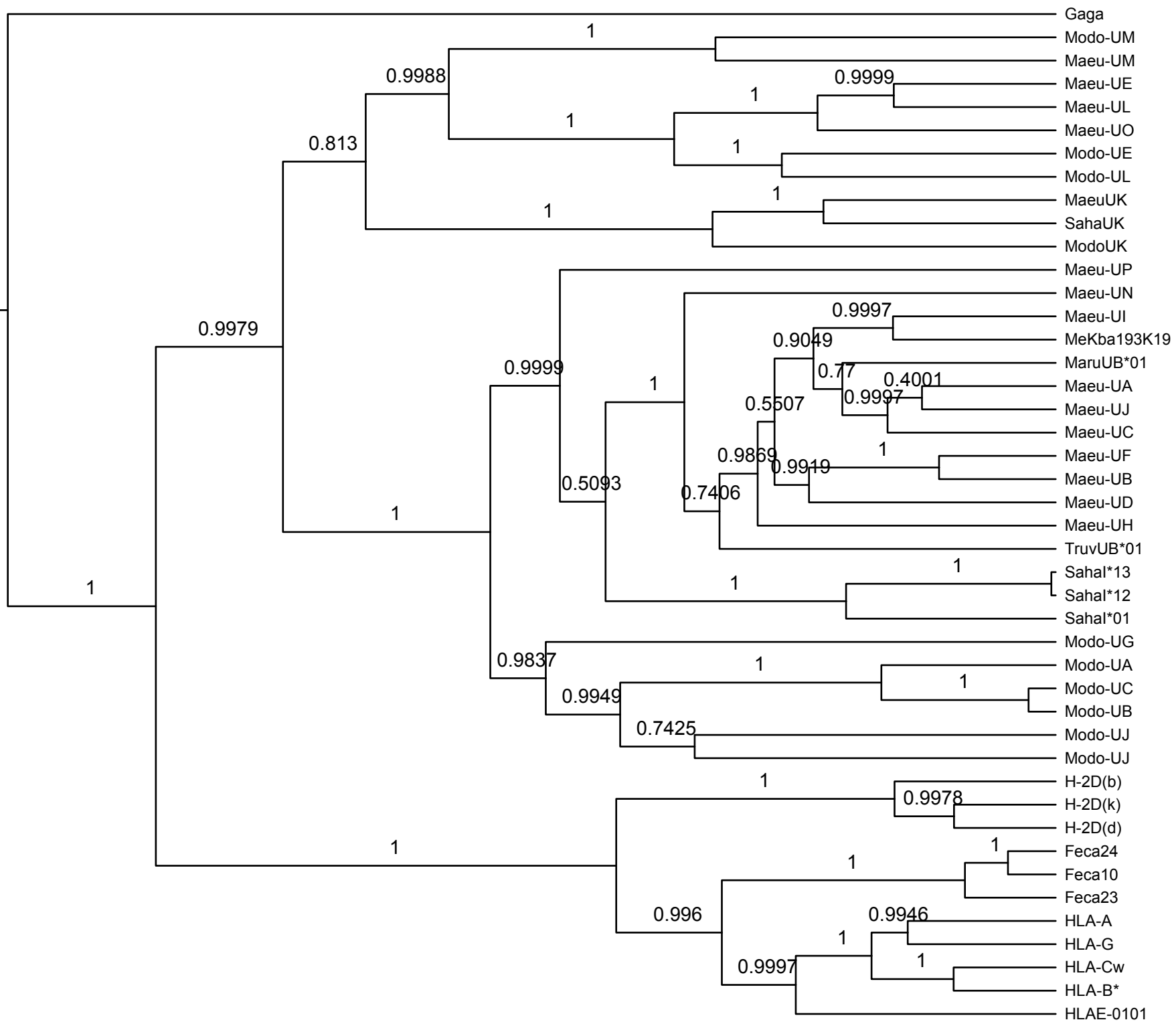

Supplement: Additional file 4 — Phylogenetic tree produced for BEAST analysis. Phylogenetic tree produced for the BEAST analysis with posterior distribution values, 1 = 100%. [file 1471-2164-10-310-S4.pdf]

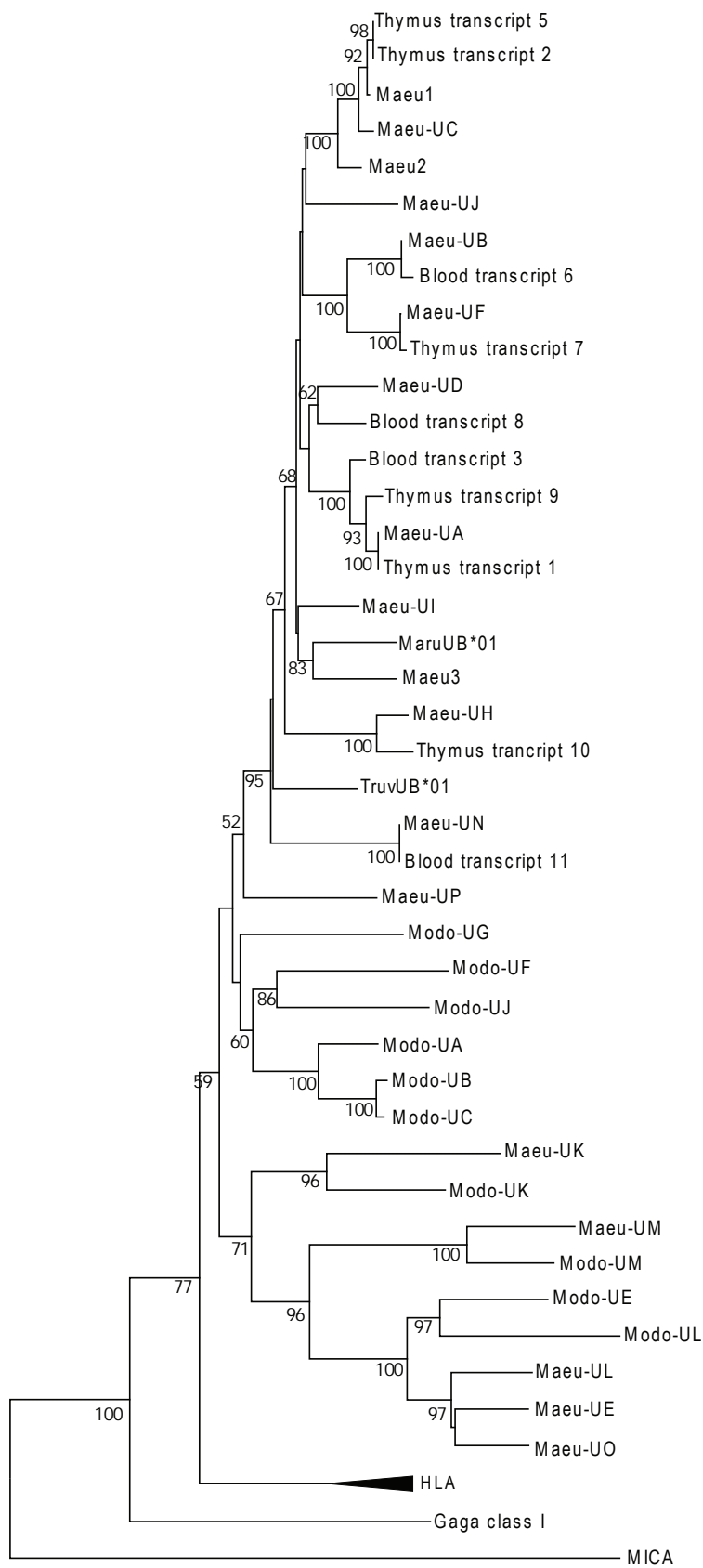

0.1

Supplement: Additional file 5 — Phylogenetic relationship between expressed class I transcripts and genomic class I genes. Neighbour joining phylogenetic tree comparing class I transcripts isolated from the tammar wallaby thymus and blood to genomic class I genes from BACs. [file 1471-2164-10-310-S5.pdf]
