# Supplementary material for: MHC-linked and un-linked class I genes in the wallaby
Source: BMC Genomics. 2009 Jul 14;10:310. doi: 10.1186/1471-2164-10-310 (PMC2719672; doi:10.1186/1471-2164-10-310)
Supplement: Additional file 2 — Amino acid alignment of tammar wallaby class I genes, opossum class I genes and HLA-A. Amino acid alignment, the following features are marked, P – residue involved in peptide binding, * – glycosylation site, c- cysteine residues forming disulphide bridges, a dash indicates a deletion in some tammar wallaby and opossum class Ib genes. [file 1471-2164-10-310-S2.pdf]

```
              -20       -10         0        10        20        30        40        50        60        70
          ....|....|....|....|....|....|....|....|....|....|....|....|....|....|....|....|....|....|....|....|
Maeu-UA   MERSLGALLLLG---TLALPETWAGPHSMRYFYTGLTRP--GFGEPRFLAVGYMDDLQFVRFDTD--RPGQREEPRAAWIEGVGQEDPEYWERNTQIYRA
Maeu-UB   .........V.R---.............L...S.AV...--.G-..QV.S......QL.SS....--S.S..M......M..ME....G....E.R.V.V
Maeu-UC   ............---.....D...........D.AV...--.S-.....S...V..Q..S.....--S...........M.RMD.........V.R.H.E
Maeu-UD   ............---.....D....S..L...D.AV...--.L......T...V..Q........--S.S..L.........A...E.G....E..NL..
Maeu-UF   ............---..V.......S..L...ASAV...--.G-...I.V...V..QL.SS..SN--S.S..M......M..M.....G....E.RNV..
Maeu-UH   ..A.V.......---..........S..L...S.AM...--.L......S...VG.QE.....S.--S.S.GL...V..M.R.E..E..F...RSG.CK.
Maeu-UI   .....CS.....---S.T..D...........H.AV.G.--.L......S...V..Q......S.--S....M......M.R....E.G...EE.RTS.G
Maeu-UJ   ............---..........S........SV...--.......FS...L..QL..G..S.--S...KQ......M.R...D......QQ.RNS..
Maeu-UN   ...HVRS.F...---..T.T....S.......AAVM.G.--ELW...YI....V..QH..L..S.--S.SL.M...V.M.QMD....G......W.S..
Maeu-UM   ..HYVLV.FF.V-VLA.T.TV....C...K..D.MVSG.RL.LE.RW.MT...V..Q.LL..HS.--YRS.TT....P...LEMPD----..LM.RHLKD
Maeu-UL   .Y--VLT.....---F.V.R.....S..LK....TMS..--.LA.T..TS.T.V..Q.VLS..S.--SDS.S....MP...H------D....E.R.S.E
Maeu-UE   .GMYVLS....S--AVVV.T..NS.S..LK...IVMSG.--.LAK...IT.T.V..QLILS..S.RDSES.ST...TP...H------D....EIRVS.E
Maeu-UK   ..PYV-LF..WA---VAV.-..KG.F..LK.LQASMSLS--.LAK...IS...V..Q......S.--NWS.....Q.P.VDKIS..---D.A..SRVV.E
Maeu-UO   ..MYMLS.....-VLS.RDTWADS.S..LK...AAMSQ.--.LAK...I..T.V..Q.VLS..S.--.ES.ST...TP...QE---I.D....E.R.S.E
Maeu-UP   ...YISS.FM..---I...T.....S..L.....SIS..--EL.KS..I....V..Q.....YS.--SSS..M....P.MD---.M.LG..KTE..HM.E
Modo-UA   ..P..LS.FV..---VV..T..R..S..L...D.AMS..--EL.DSQ.IS...V..Q......SS--SESP.M......MDK.D....N...GQ...S.S
Modo-UK   --------------------------..L...Q.SMSL.--.-RK.Q.IS...V.....ML..GN--S.N.......P.LDQMD.D---...K.SR.S.E
Modo-UM   --------------------------....ID.VV.A.RL.LE.RW.TL...V..Q.....HSN--NAS.ST.IG.P...LETPD---....EKRHLKD
HLA-A     .AVMAPRT...LLSGA...TQ....S.....F.SVS..--.R.....I....V..T......S.--AAS..M....P...---..G....DQE.RNVK.
                                          p                                                p

               80        90       100       110       120       130       140       150       160       170
          ....|....|....|....|....|....|....|....|....|....|....|....|....|....|....|....|....|....|....|....|
Maeu-UA   ATQFFRAGLETLRGYFNQSAG-GVHTFQNMYGCEVSPELRFLRGFMQYAYDGTDYIALDTETLTWTAAVPPAVSTKRKWEAERSISEGMKAYLEET-CVT
Maeu-UB   N..SY..SM.NVP........-D.....R.L..D.....T.....R......R.........Y....V.....N..HR..V.-RPA.RY.......-..Q
Maeu-UC   TA.IY.GN...A.........-......T..........T.Q...F.F............D....................A-.RA.RD......E-..E
Maeu-UD   GA.NY.....N..........-....L.R.F........T.Q...E.H....Q..L......Y.......Q...S...T..DS..A..A.......-...
Maeu-UF   IA.SY.ESM.NVP.......S-D.....R.T......D.T.Q...R......Q.........Y....V..Q.....L...V.-RPA.RR......E-..L
Maeu-UH   E.....GT..S.LS-L...S.-....I.RLH......D.T.Q...LL.....E..LV.....YR......E..HI.Q....D.N.V.RW......E-..L
Maeu-UI   NA.IY.G............V.-....I.L.S..........Q...L.F...R.......................L....DW..A.RR...V..E-..L
Maeu-UJ   NA.I..MN...A.R...H...-.....Y.S........G.Q...S......Q..L..............T...N...........A.SR......-..A
Maeu-UN   K..VY.GN.K..LS.....S.-......R.S........T.K...L.H....Q..LT...D.Y..M.......N.......D.G.V..Y.S.....-..L
Maeu-UM   -A.NC.MS.QN.HFNY...DDS...IL.RI.....FSNGS.STFYLR.G...Q.KLS..P...S.I.LDNV.LNV.F.LD.DP.LLKMWQFH.K..T.IQ
Maeu-UL   ...RY.MC.QKVST.Y.H.E.-......SLS....FSNGS.S...V......L.FL...M...L.I.GNAE.LNR..EL.E-QGLAKYW.G...GE-..Y
Maeu-UE   Q..VY.MC.QKVSA.Y.H.E.-....L.RLS....FSNRS.S...V......Q..L.......H.I.GNSG.LNH..E..E.Q.FAKYW.G...GE-..Y
Maeu-UK   TEHT.QV..QN.QV.Y...E.-K...Y.RLVA..M.SDWT.K...E.F......Q...S..L....S.....F..LNS.H..D..P..A.RQ.F...KK-.TE
Maeu-UO   ...RY.MC.QKVSS.Y.H.E.-....Y.RLS....FSNRS.S...V......M..L.......S.I.GNAG.LNN......DQR.AKYW.G.M..E-..Y
Maeu-UP   HA.DY.V...N.LR.Y...ED-....V.FF.....Y.N.T.K...E.N....Q.........Y.......E..T..H....QG..M..E.V...DE-..E
Modo-UA   NA.IT.V....I...Y...R.-.L..Y.T.I....H.DGS.RK..W.H....H......R.........D.G.EN...........A.RY......E-..Q
Modo-UK   TA.T.EV..QN.LV.Y...E.-.L..Y.RLV.....HNRM.R...E......Q...S..F...S....DI.SLNS.H...M.PN.AQRQ.T...KN-..Q
Modo-UM   -S.NCPMS.QN.HFNY...HD-....L.R......FGNGS.STF.LL.G...Q.KLS..P...N.I.SDSV.L.I.L.LD.D..PA.RW.VH.TV.-..Q
HLA-A     QS.TD.VD.G.....Y...EA-.S..I.I....D.GSDG.....YR.D....K.....NEDLRS....DMA.QI.......A-HEA.QLR...DG.-..E
                              p ***             c                p                        p  pp           p    c

              180       190       200       210       220       230       240       250       260       270
          ....|....|....|....|....|....|....|....|....|....|....|....|....|....|....|....|....|....|....|....|
Maeu-UA   WVKKYLEMGKETLMRTDPPSARVTHLTAPYGEVTLRCRAQGFYPKEISLTWLRDGEEQPQDTEFIETRPAGDGTFQKWADVHVTSGEEERYTCRVQHEGL
Maeu-UB   R.........V........T...RH...H..........D.................L.....................A.E....Q.G..........
Maeu-UC   ..........A............RH...H..........D..........................A...............A.E....Q.G..........
Maeu-UD   .L......................HIT.R..........D........K................................T.D......GK.........
Maeu-UF   ....F......A........T...RH...H..........D.........................................A.E....Q.G..........
Maeu-UH   .L.....T.......K.S...Q..RH...H..........D........I.................................VM...Q.DK...Q......
Maeu-UI   .LR........V............RH.D.H..........D.........................................A.D....Q.G..S........
Maeu-UJ   ..........A............RH...H.......W.HD................S.....................A.E..C.Q.G..........
Maeu-UN   .L..H......I............CH...S.D........D............N....S....................A.D..P.Q.GK....I.....
Maeu-UM   ALLRH..K..K..L......VK..RHISSDW....K...W....AK...I.........I....Y.....G.........IA.GMPF.N...Q...Q......
Maeu-UL   .LQ...DN.....L......VK..RHISSE..............A.............L.EM.L...............ALGML..S.QK.........
Maeu-UE   .LQR..DN.....L...L..VQ..RH.SSD..........H....S....D.........L.E..L...............A.RML..N.QK.........
Maeu-UK   .LQ....Y...M.L.AE...VQ..RH.VHE...I.......D...A...MI.........L..M.................A.RI....Q.GK.A........
Maeu-UO   .LQR...N.....L.I....V...RH.SSD...........S...A.......M.....L.E..L...............A.GML..S.QK.....R....
Maeu-UP   .LR....I...Y.N......VQ..RH...DE.........D...S.........D...L.N..................A.GI...Q.GK.A........
Modo-UA   .L....Q...DV.L..E.S....SRHSG.D...S..........A.............L.........G.........A.AMAP.Q.D..S......A.
Modo-UK   .LY....I.N...L.AG........HISHE.K.M...W.RD...AD...I.........LHN........G.........T.AMAP.Q.D..S......--
Modo-UM   .LLRH..K.....L......VQ..RHITSDS..I.K...W....A....I...N....I....Y.D...G.........A.EMPF.N...K........--
HLA-A     .LRR...N.....Q.....KTHM..HPISDH.A....W.L....A..T...Q....D.T....LV..............A.V.P....Q....H.....
              p                              c                                                c

              280       290       300       310       320       330       340
          ....|....|....|....|....|....|....|....|....|....|....|....|....|....|....
Maeu-UA   SEPLTLQWEPESSSTWLIVGGIA---AALLILIAVIAGVGIWRKKNSGVKGGDYVQAAGNDSAQGSDVSLTAKA
Maeu-UB   P.........Q..L......A..---.G..L.T..VV......R.H..G..S...P..................
Maeu-UC   P............F......A..---..I........V.WHCI.RL..GE.....P.................-
Maeu-UD   P.....................---..................R...G.....P...........I......
Maeu-UF   P.........Q..L......A..---.G-.L.T...V......R.H..G..S...P..................
Maeu-UH   ..........Q......F..S.G---.V..L.A..L........R.TDGI.....P.T.........I...V..
Maeu-UI   ................H...A.V---.G..L.........M.......G...............R..IP.....
Maeu-UJ   P.....................---V.....................G......PT...E............L
Maeu-UN   ..........Q......T.....--AGL..L....L....M.......GN.D.F..T...............RV
Maeu-UM   P...S.K...Q.A.IG...I------IFAVV....VV.AVM.K.YT..G...NCALTREK............TG
Maeu-UL   P...F.K...Q..TIG.SARV.T---.L..L.A...I..V....NIPDG.RES.TTT.S...........V..G
Maeu-UE   P...F.K.G.Q..I.G.SA.V.T---.L..L.AS..VSAV....NT.DR.R.S.TTT.SS.............-
Maeu-UK   ....A.K...Q.KY..ITL.I..-----VF..HL.....V.LKNR...G.RL.......---------------
Maeu-UO   P...F.K...Q..VIG.SA.I.T-----A.L.A...V..V...RNT.DG.R.R.TTT.ST.............G
Maeu-UP   .....MK.G.QF.Y..ITA--------VV.L...LVI..V...NR..--------------------------
Modo-UA   AQ..S.R....A..L.V...VT.---GV.VLVT..V..AV.L.RR...G...A..P..DK............T.
Modo-UK   --------------------------------------------------------------------------
Modo-UM   --------------------------------------------------------------------------
HLA-A     PK....R..LS.QP.IP...I..GLVLLGAVITGAVVAAVM..R.S.DR...S.T...SS............CK
                                                               *
```
